# Association of Cytotoxic T Lymphocyte Antigen-4 Gene Polymorphisms with Psoriasis Vulgaris: A Case-Control Study in Turkish Population

**DOI:** 10.1155/2018/1643906

**Published:** 2018-04-23

**Authors:** Hatice Gül Dursun, Hüseyin Osman Yılmaz, Recep Dursun, Sevsen Kulaksızoğlu

**Affiliations:** ^1^Department of Medical Biology, Meram Faculty of Medicine, Necmettin Erbakan University, 42080 Konya, Turkey; ^2^Department of Dermatology, Meram Faculty of Medicine, Necmettin Erbakan University, 42080 Konya, Turkey; ^3^Department of Clinical Biochemistry, School of Medicine, Baskent University, Konya, Turkey

## Abstract

Psoriasis is a common, chronic, and autoimmune skin disease in which dysregulation of immune cells, particularly T cells, is thought to play an important role in the pathogenesis. Cytotoxic T lymphocyte antigen-4 (CTLA-4) expressed only on activated T cells is an immunoregulatory molecule and plays a role in the pathogenesis of autoimmune disorders. We aimed to determine whether CTLA-4 gene polymorphisms are associated with development and/or clinical features of psoriasis vulgaris (Pv). Genotyping of SNPs (−318C>T, +49A>G, and CT60A>G) in CTLA-4 gene was performed using polymerase chain reaction-restriction fragment length polymorphism (PCR-RFLP) in 103 Pv patients and 102 controls. No statistically significant associations were detected in any of the investigated genetic models for the −318C>T polymorphism. The genotype distributions of +49A>G and CT60A>G were associated with Pv development. In haplotype analysis, while frequency of CAA haplotype was significantly higher in the control group, frequencies of CGG and CAG haplotype were significantly higher among the patients. However, all of CTLA-4 polymorphisms and haplotypes do not have an effect on severity and onset age of Pv. In conclusion, the +49A>G and CT60A>G polymorphisms may be risk factors for Pv development. Furthermore, CGG and CAG haplotypes may contribute to Pv development, while CAA haplotype may be protective against Pv.

## 1. Introduction

Psoriasis is a common inflammatory skin disease that affects approximately 125 million people globally [1]. The disease that exhibits a variable clinical presentation is characterized by lesions in the form of circular, red papules and plaques with a grey or silvery-white, dry scale. Psoriatic lesions are generally distributed symmetrically on the scalp, elbows, knees, lumbosacral area, and umbilicus [2, 3]. In addition, nail disease and/or psoriatic arthritis, which can be very painful and deforming, may develop in many patients with psoriasis [2–5]. The incidence of psoriasis in women and men is almost equal [3]. Psoriasis is associated with several comorbidities, such as Chron's disease [6, 7], cardiovascular syndrome [8, 9], metabolic syndrome [10–12], depression [13], and cancer [14, 15]. The disease leads to a serious reduction in the quality of a patient's life, because it is linked with social stigmatization, pain, discomfort, physical disability, and psychological distress [2]. Recently, psoriasis has begun to be defined as a disease spectrum or systemic disease because of abovementioned concomitant comorbidities. As a result, it requires lifelong treatment [16]. Although the molecular pathogenesis of the disease is still poorly understood, it is generally agreed that psoriasis is triggered by some environmental factors such as stress, infections, trauma, and drugs with a genetic background [17]. The common view about the molecular pathogenesis of the disease is that alterations in the complex interactions between T lymphocytes, dendritic cells, macrophages, mast cells, neutrophils, keratinocytes, cytokines, and chemokines cause psoriasis, and this wise unbalanced immune response contributes to the psoriatic process [18, 19]. Psoriasis has four major clinical phenotypes, which are distinguished by the morphological characteristics of their lesions: (i) psoriasis vulgaris, (ii) guttate psoriasis, (iii) pustular psoriasis, and (iv) erythrodermic psoriasis [20]. The most common of these clinical phenotypes is psoriasis vulgaris, responsible for 90% of all cases, and is also known as plaque psoriasis [3]. In this phenotype, the lesions are dry, sharply demarcated, oval/circular plaques and can be localized all over the body, but eventually affecting mostly the knees, elbows, lumbosacral area, intergluteal cleft, and scalp[20, 21].

Cytotoxic T lymphocyte antigen-4 (CTLA-4) is an important immunoregulatory molecule that plays a role in the maintenance of T cell homeostasis. In T cell-mediated immunological response, the interaction of MHC on antigen-presenting cell (APC) with CD28 on T cell is essential but not sufficient for T cell activation. However, the additional costimulatory factors and pathways are required for T cell activation [22, 23]. One of the costimulatory pathways is B7- (CD80/86) CD28 [22]. CD28 expressed on antigen-presenting cells by naive T cells binds to B7 (CD80/86) initiates the proliferation, differentiation, and cytokine production in T cells. Binding of CTLA-4 expressed by T cells to B7 presented on APC contributes to peripheral tolerance leading to the arrest of T cell cycle and termination of T cell activation [22, 24]. CTLA-4 acts as an inhibitor of autoimmunity, and the defects in the B7-CD28/CTLA-4 pathway may lower the threshold of autoreactive lymphocyte activation and which in turn may lead to the development of an autoimmune disease [25]. CTLA-4 molecule is encoded by the CTLA-4 gene (gene ID: 1493; OMIM^∗^123890) located on chromosome 2p33 [26]. Several polymorphisms were identified in the CTLA-4 gene. The polymorphisms reducing the CTLA-4 expression or function may cause autoimmune clonal T cell proliferation and thus the development of autoimmune diseases [27]. In fact, some association studies indicated that there is an association between several CTLA-4 gene polymorphisms and various autoimmune diseases [28–48]. Recently, it has been shown that polymorphisms of many genes that are directly or indirectly related to the immune system and/or inflammation are associated with psoriasis. These include genes such as ADAM33 (a disintegrin and metalloprotease33) [49], TLR2 and TLR4 (toll-like receptor 2 and 4) [50], MCP-1 (monocyte chemoattractant protein-1) and RANTES (regulated upon activation normal T cell expressed and secreted) [51], TNF*α* (tumor necrosis factor alpha) [52], PON1 (paraoxonase) [53], IL-4 and IL-10 (interleukins) [54], HLA [55], VEGF (vascular endothelial growth factor) [56], and ERAP (endoplazmic reticulum aminopeptidase) [57]. However, there are a few studies establishing a possible relationship between CTLA-4 gene polymorphisms and psoriasis.

In the present study, we have conducted a research on three single-nucleotide polymorphisms (SNPs) in the CTLA-4 gene, because of its possible effects on expression level or function of the CTLA-4 molecule: −318C>T (in promoter), +49A>G (in exon-1), and CT60A>G (in exon-4). With this hypothesis, our goal was (i) to investigate whether the CTLA-4 gene polymorphisms are related to the development of Pv (psoriasis vulgaris) and (ii) to detect whether the CTLA-4 gene polymorphisms have an impact on the clinical features of *P. vulgaris* such as onset age and severity. In the literature, there are few studies which observed the relationship between CTLA-4 gene polymorphisms and Pv [58–61]. Yet, there are no previous studies revealing a relationship between CTLA-4 genes −318C>T, +49A>G, and CT60A>G SNPs and the development of Pv.

## 2. Subjects and Methods

### 2.1. Research Population

103 unrelated Turkish Pv patients were selected for the experimental group, and 102 unrelated healthy Turkish people were selected for the control group. Psoriasis vulgaris patients (66 female/37 male; mean age ± SD: 37.83 ± 16.83) were recruited from a dermatologic clinic. The patients with other chronic and autoimmune diseases or cancer were excluded from the study. The control group (58 female/44 male; mean age ± SD: 37.23 ± 16.77) was formed with healthy individuals who did not have cancer, psoriasis, and other autoimmune diseases and did not have a family history of these diseases. The patients and control subjects were matched according to their gender and age. Severity of psoriasis was assessed with Psoriasis Area and Severity Index (PASI), ranging from 0 (no disease) to 72, with higher scores indicating the severity of disease [62]. To determine the association of CTLA-4 gene polymorphisms with the clinical features of Pv, the patients were divided into two groups according to the severity of disease (PASI < 12 group and PASI ≥ 12 group) and then assigned into two groups according to the onset of disease (early-onset group: <40 age and late-onset group: ≥40 age) (Table 1).

This study was conducted in accordance with the Declaration of Helsinki principles and was approved by the Ethics Committee of Meram Medical Faculty (number 2010/138). Informed consent was obtained from all the participants before the study.

### 2.2. Genotyping

Peripheral blood sample was taken from each patient and control subject collected in tubes containing EDTA and stored at −20°C before DNA isolation. Genomic DNA was extracted from the blood sample using the QIAamp DNA Blood Mini Kit (Qiagen, Hilden, Germany). Genotyping of the CTLA-4 gene −318C>T (rs5742909), +49A>G (rs231775), and CT60A>G (rs3087243) was carried out by the polymerase chain reaction-restriction length polymorphism (PCR-RFLP) using *Mse*II, *Bbv*I, and *Nco*I enzymes (New England BioLabs, Hitchin, UK). PCR reactions were performed with mixtures consisting of 0.2 *μ*g genomic DNA, 5 *μ*l ammonium buffer, 4.5 *μ*l MgCl_2_, 20 pmol of each primer, 5 unit Taq polymerase, and double-distilled H_2_O up to final volume of 50 *μ*l. The primers were designed according to the complete CTLA-4 gene sequence derived from NCBI Sequence Viewer (http://www.ncbi.nlm.nig.gov/). PCR was carried out with denaturation at 95°C for 5 minutes, followed by 35 cycles of 45 seconds at 94°C, 45 seconds at 55°C, and 45 seconds at 72°C and finally 10 minutes at 72°C. The PCR products were digested using 10 U of *Mse*II, 6 U of *Bbv*I, and 10 U of *Nco*I enzymes and then electrophoresed on 2.5% agarose gel, stained with ethidium bromide, and evaluated. The primers used for PCR, conditions for digestion, products of digestion, and genotypes determined according to the products of digestion are listed in Table 2.

### 2.3. Statistical Analysis

The SPSS 13.0 package programme was used for data analysis. Comparisons of the distributions of allele and genotype frequencies were performed by Pearson's chi-squared test. The deviation from the Hardy-Weinberg equilibrium was tested using chi-square analysis. To test the association between Pv and CTLA-4 polymorphisms, logistic regression analysis was performed according to five inheritance models (codominant 1, codominant 2, dominant, recessive, and log-additive). Odds ratios (OR), 95% confidence intervals (CI), and *p* values were determined using SNPStats (http://bioinfo.iconcologia.net/index.php?module=Snpstats) and SPSS 13.0 program. The linkage disequilibrium (LD) blocks and haplotypes were estimated using Haploview version 4.2 (http://www.broadinstitute.org/scientific-community/science/programs/medical-and-population-genetics/haploview). *p* values less than 0.05 were considered significant.

## 3. Results

### 3.1. Genotype Analysis and Association of SNPs with Pv

Table 3 shows the genotype and allele frequencies of CTLA-4 polymorphisms (−318C>T, +49A>G, and CT60A>G) in Pv patients and the control group. The genotype distributions of the examined SNPs were consistent with the Hardy-Weinberg equilibrium (HWE) (Table 3).

In multiple logistic regression analysis, −318C>T SNP was not associated with the development of Pv (*p* > 0.05 for all genetic models and T allele frequency). However, +49A>G and CT60A>G SNPs were associated with Pv. The disease-related risk was observed in the codominant 1 model (OR = 0.57, *p* = 0.04), dominant model (OR = 0.54, *p* = 0.03), and log-additive model (OR = 0.62 and *p* = 0.03) for +49A>G and in the codominant 2 model (OR = 0.29, *p* = 0.004) and recessive model (OR = 1.33, *p* = 0.001) for CT60. In addition, G allele (minor allele) frequencies of both +49A>G and CT60A>G SNPs were higher in the Pv patient (31% for +49A>G and 55% for CT60A>G) than in the control group (21% for +49A>G and 40% for CT60A>G) (OR = 0.59, *p* = 0.02 for +49A>G and OR = 0.54, *p* = 0.002 for CT60A>G).

### 3.2. Genotype Analysis and Association of SNPs with Clinical Features of Pv

Tables 4 and 5 present the genotype and allele frequencies of CTLA-4 SNPs in clinical subgroups of Pv (onset age of disease and severity of disease). None of the examined SNPs showed no association with onset age and severity of Pv (for all genetic models). The genotype and allele frequencies of examined SNPs did not differ between the early group and late group (Table 4) and PASI < 12 group and PASI ≥ 12 group (Table 5). The results indicated that −318C>T, +49A>G, and CT60A>G SNPs have no effect on the onset age and severity of Pv.

### 3.3. Linkage Disequilibrium and Haplotype Analysis

We estimated the linkage disequilibrium (LD) block by using Haploview version 4.2. The LD block was strongly made between −318C>T and +49A>G (*D*' = 0.999 and *r*^2^ = 0.043), −318C>T and CT60A>G (*D*' = 0.999 and *r*^2^ = 0.141), and +49A>G and CT60A>G (*D*' = 1.000 and *r*^2^ = 0.383). In haplotype analysis which was performed to investigate the association between the haplotypes of LD block SNPs and Pv, four major haplotypes were detected which are CAA, CGG, TAG, and CAG (Table 6). The frequencies of these haplotypes were 0.532, 0.253, 0.110, and 0.103, respectively. A significantly higher frequency of CAA haplotype was found in controls (0.603) than in Pv patients (0.461, *p* = 0.004). In contrast, significant increases in the frequencies of CGG and CAG haplotypes were observed in patients (0.306 and 0.146, resp.) compared to healthy individuals in the control group (0.201 and 0.059, resp.; *p* = 0.015 and *p* = 0.004). These results suggest that while the CAA haplotype may have a protective effect on the development of Pv, the CGG and CAG haplotypes may be associated with the development of Pv. In haplotype analysis which was performed to investigate the association between the haplotypes and the clinical subgroups of Pv, three major haplotypes were detected which are AA, GG, and AG (Table 7). The frequencies of these haplotypes were 0.461, 0.306, and 0.233, respectively. Considering the onset age of Pv, the frequencies of these haplotypes did not differ between the early group and the late group (*p* = 0.467, *p* = 0.434, and *p* = 0.243, resp.). There was no significant difference between the PASI < 12 group and the PASI ≥ 12 group with respect to the frequencies of AA, GG, and AG haplotypes (*p* = 0.069, *p* = 0.373, and *p* = 0.243, resp.).

## 4. Discussion

Psoriasis is an inflammatory disease which is characterized by keratinocyte proliferation and activated T cell accumulation [63]. The incidence of psoriasis in women and men is almost equal [3]. However, in our study, the number of female patients (66) was significantly higher than the number of male patients (37). This situation is entirely coincidental and only results from the fact that the number of female patients who applied to the clinic during the study period was more than the number of male patients. Probably, the number of female patients and male patients would be close if the study period was extended a little longer or the number of patients could be increased.

Although its pathogenesis has not been well understood, psoriasis bears many features of a T cell-mediated autoimmune disease. It reveals a strong HLA association [64]. Since CTLA-4 regulates T cell activation and the proliferation through a negative feedback, the CTLA-4 gene is considered to be a candidate gene for T cell-mediated autoimmune disease. Hence, in this study, we aimed to investigate the possibility of an association between this candidate gene and Pv, which is defined as an autoimmune disease. In the present study, −318C>T, +49A>G, and CT60 polymorphisms were studied to evaluate their contributions to the pathogenesis of Pv, focusing on their potential effects on the activity and function of the CTLA-4 molecule. In fact, it has been suggested that −318C>T polymorphism is an effective promoter activity of the CTLA-4 gene and change transcription of CTLA-4 gene [65]. +49A>G polymorphism is located in the leader sequence which is important in the binding of the CTLA-4 molecule to B7.1 (CD80). CT60A>G polymorphism is considered to affect the alternative splicing and soluble CTLA-4 production [66].

Our data displayed no association between −318C>T SNP and the development of Pv. There were no differences in genotype and allele frequencies between the patient group and the control group. Likewise, Łuszczek et al. [60] found an association between polymorphism and Pv in their study. It has been also indicated that the association of −318C>T polymorphism with other autoimmune disorders supports our hypothesis. The association of −318C>T polymorphism with other autoimmune diseases such as spondyloarthropathy [67], pemphigus foliaceus [30], multiple sclerosis [38, 68], Behçet's disease [35], systemic lupus erythematosus [37, 69], Hashimoto's thyroiditis [41, 44], ankylosing spondylitis [40], and Graves' disease [70] supports our findings in which the researchers did not find any significant relationship between −318C>T and other diseases; however, an association between −318C>T polymorphism and other autoimmune disorders was found. The association of −318C>T polymorphism with childhood Graves' disease was reported in a Chinese population [34]. In a study on a Chinese population, a significant relationship was found between −318C>T polymorphism and rheumatoid arthritis [47, 71]. In the Italian systemic sclerosis patients, an association was found between −318C>T polymorphism and the susceptibility to develop systemic sclerosis [33].

+49A>G SNP is a CTLA-4 gene polymorphism which is probably the most widely studied and most commonly associated with autoimmune disorders and cancers. In our study, +49A>G polymorphism indicated a strong relationship with Pv in terms of minor allele frequency (OR = 0.59, 95% CI = 0.38–0.92, *p* = 0.02), codominant 1 model (OR = 1.54, 95% CI = 0.48–5.01 *p* = 0.04), dominant genetic model (OR = 0.54, 95% CI = 0.40–0.96, *p* = 0.03), and log-additive genetic model (OR = 0.62, 95% CI = 0.40–0.96, *p* = 0.03). In addition, +49A>G SNP might contribute to the risk of Pv development and G allele might be a risk factor in Pv development. This SNP causes substitution of threonine at position 17 to alanine in the CTLA-4 protein [72]. It has been postulated that this amino acid substitution may affect T cell activation by changing the posttranslational modification and ability of CTLA-4 to bind with B7.1 (CD80) [73]. Various studies have revealed that the +49G allele leads to decreased expression of CTLA-4 compared to +49A allele [27, 74]. Our findings are probably related to +49A>G SNP and may be explained by the inability of CTLA-4 to bind to B7 and/or by decreasing of CTLA-4 expression. Furthermore, decreased expression and/or broken binding with B7 in CTLA-4 may contribute to the pathogenesis of Pv by changing the T cell response. The findings of this study are inconsistent with the results of Tsunemi et al. [58], Kim et al. [59], and Łuszczek et al. [61] who evaluated the association between +49A>G polymorphism and Pv. Łuszczek et al. [61] studied on 141 Pv patients recruited from a Polish population and found that the allele and genotype distributions of +49A>G polymorphism are similar for the patients in the experimental group and healthy individuals in the control group. In the studies with Japanese [58] and Korean [59] populations, no association was reported between polymorphism and Pv. However, the results of some studies examining the relationship of +49A>G polymorphism with other autoimmune disorder, but not with Pv, are consistent with the results of our study. These studies revealed the association of +49A>G polymorphism with Graves' disease [27–29, 34, 70], rheumatoid arthritis [39], and ankylosing spondylitis [40]. On the other hand, it has been shown that there is no relation between +49A>G polymorphism and several autoimmune diseases such as rheumatoid arthritis [43, 71], Behçet's disease [35], vitiligo [75], systemic lupus erythematosus [37, 69], systemic sclerosis [33], spondyloarthropathy [67], ankylosing spondylitis [36, 40], pemphigus foliaceus [30], multiple sclerosis [38, 68], primary Sjögren syndrome [31], and ulcerative colitis [48].

In the present study, we observed a strong association between CT60A>G polymorphism and Pv in terms of codominant 2 (OR = 0.29, 95% CI = 0.13–0.64, *p* = 0.004), recessive (OR = 1.33, 95% CI = 0.17–0.60, *p* = 0.001), and minor allele frequency (OR = 0.54, 95% CI = 0.37–0.80, *p* = 0.002). Allele G appears to be a risk factor for the development of Pv. Łuszczek et al. [61] observed no difference in allele and genotype distributions of CT60A>G polymorphism between Pv patients and control subjects. This SNP is located in 3′ UTR (untranslated region) of the CTLA-4 gene and is supposed to affect the proportion of soluble isoform of CTLA-4 (sCTLA-4) to membrane-bound CTLA-4 (mCTLA-4). sCTLA-4 isoform is generated through alternative splicing of CTLA-4 mRNA. It has been previously suggested that the G allele on position +6230 (CT60G) may decrease sCTLA-4 transcript up to 50% [66]. Furthermore, we also observed higher frequencies of G allele and GG genotype in Pv patients than the control group. It is assumed G allele causes a decrease in CTLA-4 expression and deterioration of the balance between sCTLA-4/mCTLA-4 by blocking the alternative splicing of CTLA-4 mRNA. Chong et al. [34] have suggested that CT60A>G polymorphism plays a role in susceptibility to childhood Graves' disease. Kavvoura et al. [32] have discovered that polymorphism can be an important marker of genetic risk in Graves' disease and Hashimoto thyroiditis. Furthermore, it has also been suggested that CT60A>G polymorphism leads to the susceptibility of vitiligo [75] and ankylosing spondylitis [40].

There are several reasons that could explain these controversial results among different studies: (i) studied populations have different ethnic features, (ii) studied populations have different sizes, and (iii) studied autoimmune disorders have already a multifactorial nature. In this study, −318C>T, +49A>G, and CT60A>G polymorphisms were selected because they can play a role on Pv pathogenesis by altering the promoter activity and transcription efficiency (for −318C>T), by altering T cell activation through posttranslational modification (for +49A>G), and by affecting the alternative splicing and production of CTLA-4 isoforms (for CT60A>G). Although our population size was relatively small, we believe that our results will contribute to meta-analysis studies which have aimed at understanding the role of CTLA-4 on the pathogenesis of Pv.

## 5. Conclusions

To conclude, our data suggest that while there seems to be no correlation between −318C>T polymorphism and the development of Pv, +49A>G and CT60A>G polymorphisms may be associated with the development of Pv. In addition, our results present that none of the studied polymorphisms were related with the clinical features of Pv such as severity and onset age of disease. In performed haplotype analysis, CGG and CAG haplotypes were found to be the risk factor for the development of Pv, while CAA haplotype was found to be a protective haplotype for Pv. The haplotypes showed no association with severity and onset age of Pv. As a result, all of these findings suggest that +49A>G and CT60A>G polymorphisms of the CTLA-4 gene may play a role in the pathogenesis of Pv.

## Figures and Tables

**Table 1:** Characteristics of the study population.

	Patients				Control
Total number (*n*)	103				102

Female/male (*n*)	66/37				58/44

Age (mean ± SD (year))	37.83 ± 16.83				37.23 ± 16.77

Other features	With *P. vulgaris*				Healthy
	Unrelated				Unrelated
	Without cancer history				Without cancer history
	Without other autoimmune disorders and other chronic diseases				

Subgroups	According to the age of onset		According to the age of severity		
	Early < 40 age (*n*)	Late ≥ 40 age (*n*)	PASI < 12 (*n*)	PASI ≥ 12 (*n*)	
	88	15	59	44	

**Table 2:** Primers, conditions for digestion, products of digestion, and genotypes according to products of digestion.

SNP	Primers	Amplicon (bp)	RE	Temperature and duration of digestion	Products of digestion (bp) and genotypes
−318C>T	F: 5′-AAATGAATTGGACTGGATGGT-3′ R: 3′-TTACGAGAAAGGAAGCCGTG-5′	247	*Mse*II	37°C, overnight	CC: 247 CT: 20, 95, 132, 247 TT: 20, 95, 132

+49A>G	F: 5′-TTGCTCTACTTCCTGAAGACCTGAA-3′ R: 3′-AAAGTCTCACTCACCTTTGCAGAAG-5′	166	*Bbv*I	37°C, overnight	AA: 166 AG: 76, 90, 166 GG: 76, 90

CT60 A>G	F: 5′-CAC CACTATTTGGGATATACC-3′ R: 3′-AGGTCTATATTTCAGGAAGGC-5′	216	*Nco*I	37°C, overnight	AA: 20, 196 AG: 20, 196, 216 GG: 216

**Table 3:** Genotype and allele frequencies of CTLA-4 gene polymorphisms in Pv patients and control and the association of these polymorphisms with Pv.

SNP	Genotype/allele	Cases *n* (%)	Controls *n* (%)	HWE *p* (cases)	HWE *p* (controls)	Model	OR (95% CI)	*p*
rs5742909	CC	86 (0.83)	75 (0.74)	0.79	0.44	Codominant 1	1.84 (0.92–3.70)	0.85
(−318C>T)	CT	16 (0.16)	26 (0.25)			Codominant 2	1.08 (0.07–17.89)	0.22
	TT	1 (0.01)	1 (0.01)			Dominant	1.80 (0.91–3.56)	0.09
						Recessive	0.95 (0.06–15.67)	0.97
	C	188 (0.91)	176 (0.86)			Log-additive	1.67 (0.88–3.17)	0.11
	T	18 (0.09)	28 (0.14)			Minor allele	1.66 (0.88–3.11)	0.11

rs231775	AA	51 (0.50)	66 (0.65)	0.53	0.31	Codominant 1	0.57 (0.31–1.04)	0.04^a^
(+49A>G)	AG	41 (0.39)	30 (0.29)			Codominant 2	0.43 (0.15–1.25)	0.09
	GG	11 (0.11)	6 (0.06)			Dominant	0.54 (0.31–0.95)	0.03^a^
						Recessive	0.53 (0.19–1.50)	0.22
	A	143 (0.69)	162 (0.79)			Log-additive	0.62 (0.40–0.96)	0.03^a^
	G	63 (0.31)	42 (0.21)			Minor allele	0.59 (0.38–0.92)	0.02^a^

rs3087243	AA	25 (0.24)	36 (0.35)	0.11	0.65	Codominant 1	0.81 (0.42–1.55)	0.06
(CT60A>G)	AG	43 (0.42)	51 (0.50)			Codominant 2	0.29 (0.13–0.64)	0.004^a^
	GG	35 (0.34)	15 (0.15)			Dominant	0.58 (0.17–0.66)	0.07
						Recessive	1.33 (0.17–0.66)	0.001^a^
	A	93 (0.45)	123 (0.6)			Log-additive	1.38 (0.80–2.40)	0.25
	G	113 (0.55)	81(0.40)			Minor allele	0.54 (0.37–0.80)	0.002^a^

SNP: single-nucleotide polymorphism; HWE: Hardy-Weinberg equilibrium; OR: odds ratio; CI: confidence interval. ^a^Statistically significant values (*p* < 0.05). Codominant 1: major allele homozygotes versus heterozygotes; codominant 2: major allele homozygotes versus minor allele homozygotes; dominant: major allele homozygotes versus heterozygotes + minor allele homozygotes; recessive: major allele homozygotes + heterozygotes versus minor allele homozygotes; log-additive: major allele homozygotes versus heterozygotes versus minor allele homozygotes.

**Table 4:** Genotype and allele frequencies of CTLA-4 gene polymorphisms in the early-onset subgroup and late-onset subgroup and the association of these polymorphisms with onset age of Pv.

SNP	Genotype/allele	Early onset *n* (%)	Late onset *n* (%)	HWE *p* (early)	HWE *p* (late)	Model	OR (95% CI)	*p*
rs5742909	CC	74 (0.84)	12 (0.8)	0.62	0.67	Codominant 1	1.42 (0.35–5.75)	0.76
(−318C>T)	CT	13 (0.15)	3 (0.2)			Codominant 2	0.00 (NA)	
	TT	1 (0.01)	0 (0.0)			Dominant	1.32 (0.33–5.30)	0.7
						Recessive	0.00 (NA)	
	C	188 (0.91)	176 (0.86)			Log-additive	1.19 (0.33–4.30)	0.8
	T	18 (0.09)	28 (0.14)			Minor allele	1.19 (0.32–4.39)	0.11

rs231775	AA	45 (0.51)	6 (0.4)	0.49	0.98	Codominant 1	1.54 (0.48–5.01)	0.04
(+49A>G)	AG	34 (0.39)	7 (0.47)			Codominant 2	1.67 (0.29–9.62)	0.72
	GG	9 (0.1)	2 (0.13)			Dominant	1.57 (0.52–4.78)	0.42
						Recessive	1.35 (0.26–6.97)	0.73
	A	124 (0.7)	19 (0.63)			Log-additive	1.35 (0.62–2.98)	0.45
	G	52 (0.3)	11 (0.37)			Minor allele	1.38 (0.61–3.10)	0.43

rs3087243	AA	24 (0.27)	1 (0.07)	0.06	0.13	Codominant 1	2.07 (0.59–7.30)	0.45
(CT60A>G)	AG	35 (0.4)	10 (0.67)			Codominant 2	0.30 (0.03–2.89)	0.08
	GG	29 (0.33)	4 (0.27)			Dominant	1.35 (0.40–4.61)	0.62
						Recessive	0.19 (0.02–1.53)	0.06
	A	93 (0.53)	18 (0.6)			Log-additive	0.77 (0.36–1.63)	0.49
	G	83 (0.47)	12 (0.4)			Minor allele	1.34 (0.61–2.94)	0.47

SNP: single-nucleotide polymorphism; HWE: Hardy-Weinberg equilibrium; OR: odds ratio; CI: confidence interval.

**Table 5:** Genotype and allele frequencies of CTLA-4 gene polymorphisms in PASI < 12 and PASI ≥ 12 and the association of these polymorphisms with the severity of Pv.

SNP	Genotype/allele	PASI < 12 *n* (%)	PASI ≥ 12 *n* (%)	HWE *p* (PASI < 12)	HWE *p* (PASI ≥ 12)	Model	OR (95% CI)	*p*
rs5742909	CC	37 (0.84)	49 (0.83)	0.25	0.48	Codominant 1	1.26 (0.42–3.78)	0.39
(−318C>T)	CT	6 (0.14)	10 (0.17)			Codominant 2	0.00 (NA)	
	TT	1 (0.02)	0 (0.0)			Dominant	1.08 (0.38–3.10)	0.89
						Recessive	0.00 (NA)	
	C	188 (0.91)	176 (0.86)			Log-additive	0.93 (0.35–2.43)	0.88
	T	18 (0.09)	28 (0.14)			Minor allele	0.93 (0.35–2.45)	0.88

rs231775	AA	23 (0.52)	28 (0.47)	0.84	0.36	Codominant 1	1.05 (0.46–2.40)	0.04
(+49A>G)	AG	18 (0.41)	23(0.39)			Codominant 2	2.19 (0.52–9.22)	0.53
	GG	3 (0.07)	8 (0.14)			Dominant	1.21 (0.55–2.65)	0.63
						Recessive	2.14 (0.53–8.60)	0.26
	A	64 (0.73)	79 (0.67)			Log-additive	1.30 (0.72–2.34)	0.39
	G	24 (0.27)	39 (0.37)			Minor allele	1.32 (0.72–2.41)	0.37

rs3087243	AA	13 (0.3)	12 (0.2)	0.79	0.23	Codominant 1	0.50 (0.19–1.28)	0.08
(CT60A>G)	AG	35 (0.4)	24 (0.41)			Codominant 2	0.40 (0.14–1.18)	0.19
	GG	10 (0.23)	23 (0.39)			Dominant	0.46 (0.19–1.11)	0.08
						Recessive	0.61 (0.25–1.51)	0.28
	A	41 (0.47)	70 (0.59)			Log-additive	0.63 (0.37–1.07)	0.09
	G	47 (0.53)	48 (0.41)			Minor allele	1.67 (0.96–2.92)	0.07

SNP: single-nucleotide polymorphism; HWE: Hardy-Weinberg equilibrium; OR: odds ratio; CI: confidence interval.

**Table 6:** Haplotype distribution belongs to CTLA-4 polymorphisms between Pv patients and control.

Haplotype	Frequency	Case/control ratios (frequency)	Chi-square	*p*
CAA	0.532	0.461, 0.603	8.274	0.004^a^
CGG	0.253	0.306, 0.201	5.959	0.015^a^
TAG	0.110	0.087, 0.132	2.12	0.145
CAG	0.103	0.146, 0.059	8.379	0.004^a^

Haplotypes were constructed in the following order: −318C>T (rs5742909)/+49A>G (rs231775)/CT60A>G (rs3087243). ^a^Statistically significant values (*p* < 0.05).

**(a):** 

Haplotype	Frequency	Late/early ratios (frequency)	Chi-square	*p*
AA	0.461	0.472, 0.400	0.529	0.467
GG	0.306	0.295, 0.367	0.612	0.434
AG	0.233	0.233, 0.263	1.364	0.243

**(b):** 

Haplotype	Frequency	PASI < 12/PASI ≥ 12 ratios (frequency)	Chi-square	*p*
AA	0.461	0.534, 0.407	3.288	0.069
GG	0.306	0.273, 0.331	0.793	0.373
AG	0.233	0.193, 0.263	1.364	0.243

Haplotypes were constructed in the following order: +49A>G (rs231775)/CT60A>G (rs3087243).
